# Canine ovarian gonadoblastoma with dysgerminoma overgrowth: a case study and literature review

**DOI:** 10.1186/s13048-019-0561-x

**Published:** 2019-09-23

**Authors:** Ana R. Flores, João Lobo, Francisco Nunes, Alexandra Rêma, Paula Lopes, Luís Carvalho, Carla Bartosch, Irina Amorim, Fátima Gärtner

**Affiliations:** 1 Department of Pathology and Molecular Immunology of the Institute of Biomedical Sciences Abel Salazar (ICBAS), University of Porto, Rua Jorge Viterbo Ferreira nr.228, 4050-313 Porto, Portugal; 2 Institute of Molecular Pathology and Immunology of the University of Porto (IPATIMUP), Rua Dr. Roberto Frias s/n, 4200-465 Porto, Portugal; 3 Center of Animal and Veterinary Sciences (CECAV), University of Trás-os-Montes e Alto Douro (UTAD), Quinta dos Prados, apartado 1013, 5001-801 Vila Real, Portugal; 4 Department of Pathology, Portuguese Oncology Institute of Porto (IPO Porto), Rua Dr. António Bernardino de Almeida, 4200-072 Porto, Portugal; 5 Cancer Biology and Epigenetics Group, Research Center (CI-IPOP) of Portuguese Oncology Institute of Porto (IPO Porto), Rua Dr. António Bernardino de Almeida, 4200-072 Porto, Portugal; 6 Epidemiology Research Unit, Institute of Public Health of the University of Porto (ISPUP), Rua das Taipas n°135, 4050-600 Porto, Portugal; 7 Marinha Grande Veterinary Hospital, Rua D. João Pereira Venâncio nr. 7, 2430-291 Marinha Grande, Portugal; 8 Institute for Research and Innovation in Health, (i3S), University of Porto, Rua Alfredo Allen, 4200-135 Porto, Portugal

**Keywords:** Bitch, Ovary, Gonadoblastoma, Dysgerminoma, Sex cord-stromal tumour, Immunohistochemistry, Karyotype

## Abstract

**Background:**

Gonadoblastoma (GB) is a rare mixed germ cell-sex cord-stromal tumour, first described in humans, commonly found in dysgenetic gonads of intersex patients that have a Y chromosome. However, this entity in not recognized in the WHO classification of tumours of genital system of domestic animals. Herein, we describe a case of ovarian gonadoblastoma with proliferation of dysgerminoma and sex cord-stromal tumour components, in a phenotypically and cytogenetically normal bitch.

**Case presentation:**

A 17-year-old cross-breed bitch had a firm, grey-white multinodular mass in the left ovary. The tumour was submitted to histopathological examination and Y chromosome detected through karyotype analysis and PCR studies. Microscopically, the ovary was almost replaced by an irregular neoplasm composed of three distinct, intermixed elements: dysgerminoma, mixed germ cell-sex cord-stromal tumour resembling human GB and a proliferative sex cord-stromal tumour component. The germ cells of gonadoblastoma and dysgerminoma components were immunoreactive for c-KIT. Sex cord-stromal cells of gonadoblastoma were immunoreactive for α-inhibin. The sex cord-stromal tumour was immunoreactive for AE1/AE3, occasionally for α-inhibin and negative for epithelial membrane antigen (EMA). The karyotype was 78, XX and PCR analysis confirmed the absence of the Y chromosome.

**Conclusion:**

Based on these findings, a diagnosis of gonadoblastoma with proliferation of dysgerminoma and sex cord-stromal tumour was made. This is the first case of ovarian gonadoblastoma in a female dog.

**Electronic supplementary material:**

The online version of this article (10.1186/s13048-019-0561-x) contains supplementary material, which is available to authorized users.

## Background

Ovarian tumours are uncommon in dogs presumably due to the high prevalence of elective ovariohysterectomy (OVH). They represent 0.5–1.2% of all canine neoplasms and are commonly found in old and multiparous bitches or in bitches with ovarian remnant syndrome [1, 2].

**Fig. 1:**
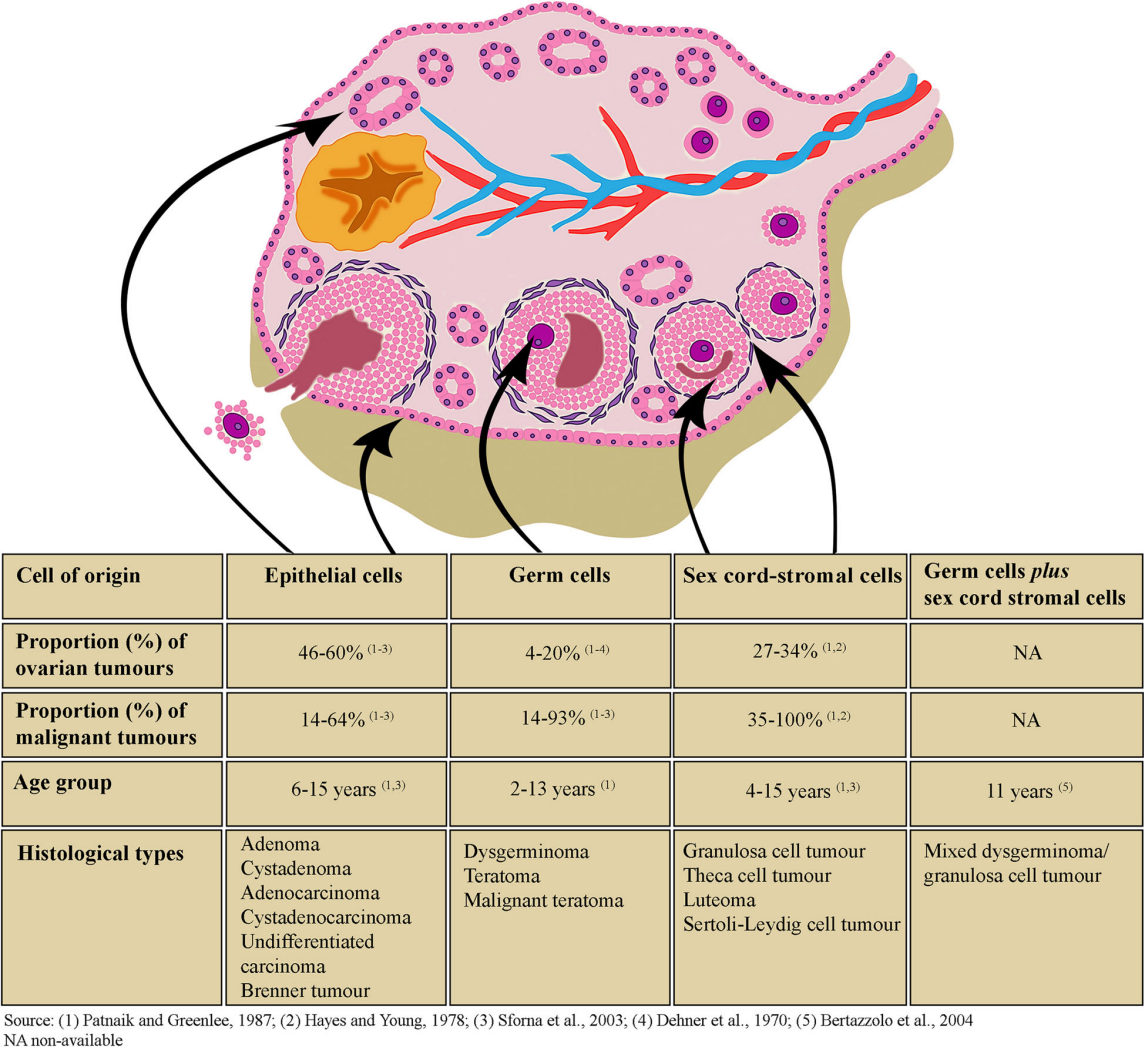
Schematic illustration of canine ovarian neoplasms. Derivation of various ovarian neoplasms and some data on their frequency and age distribution

Canine ovarian tumours are considered to originate from three ovarian cell types with distinct embryologic origins: (1) epithelial cells (from the surface, subsurface or *rete ovarii* epithelial structures); (2) germ cells and (3) sex cord-stromal cells (Fig. 1). They can also arise from nongonadal tissues around the ovaries [1, 3–6]. Combinations of more than one of the three traditional lineages have been described in ovarian lesions of domestic animals [7–9]; however, these must be distinguished from two distinct neoplastic entities, collision tumours and tissue histological variations within a single tumour [10]. Ovarian tumours composed of a mixture of germ cells and sex cord-stromal cells are rare in both humans and animals [7, 11, 12]. According to the human World Health Organization (WHO) classification of tumours of the genital system, these lesions are subclassified into gonadoblastoma (GB) or mixed germ cell-sex cord-stromal tumour (MGCSCST) [12].

Gonadoblastoma is a tumour that usually occurs in the dysgenetic gonads of intersex patients that have a Y chromosome; nevertheless, it has also been reported in normal gonads of patients without sex chromosomal abnormalities [12]. Histologically, it is composed of discrete cellular nests of germ cells intimately admixed with sex cord derivates, the later arranged in 3 typical patterns: (1) coronal (lining the periphery of the nest); (2) follicular (surrounding individual or collections of germ cells similar to the way that follicular epithelium surrounds the ovum of a primary follicle); or (3) Call-Exner-like (surrounding small round hyaline bodies, present within the cellular nests) [12]. Deposition of hyaline basement membrane material and calcification are common features within these neoplasms. By itself, it is clinically benign; however, it usually progresses to an invasive dysgerminoma or another type of malignant germ cell tumour [12].

MGCSCST arises in patients who have no evidence of gonadal dysgenesis or chromosomal abnormalities. It is less frequently associated with malignant germ cell tumours when compared to GB [12]. Generally, it lacks the discrete nested pattern of GB, hyaline bodies are uncommon, and calcification is absent. Within MGCSCSTs, sex cord components usually exhibit proliferative activity and the germ cells are thought to be non-neoplastic. In the WHO classification of tumours of domestic animals, only MGCSCSTs are referred as occurring in the male genital system [13].

Mixed tumours histologically resembling human GB have been rarely described in veterinary literature, most occurring in the testes instead of ovaries [7, 11, 14–16]. To the best of our knowledge, only two cases arising from the ovary were reported, one consisting of a mixed dysgerminoma/granulosa cell tumour in a bitch [7] and another of a bilateral GB in a female Lesser Galago [11]. The present communication describes an unprecedented case of ovarian GB with proliferation of dysgerminoma and sex cord-stromal neoplastic components, in a phenotypically and cytogenetically normal bitch.

## Case presentation

A 17-year-old cross-breed bitch (Labrador X German shepherd) was attended in Marinha Grande Veterinary Hospital Emergency Department after being hit by a car. At physical examination the animal was alert and responsive, with body temperature of 38.5° and normal capillary repletion time (CRT). Cardiac and pulmonary auscultation was normal. Musculoskeletal examination revealed a painful and non-weight bearing left hind limb. The animal had history of irregular oestrous cycles with a successful pregnancy in 2010. Hemogram and serum biochemistry were within the reference ranges. Radiographic studies revealed a luxation of the left coxofemoral joint and the animal was submitted to corrective orthopaedic surgery. At the owner’s request, an elective OVH was also performed. The excised ovaries and uterus were fixed overnight in 10% buffered formalin and sent for histopathological evaluation. An EDTA blood sample was collected for chromosome analysis.

### Pathologic findings

Grossly, the left ovary measured 5.0 × 4.5 × 3.0 cm and had heterogeneous colour; the cut surface presented a 2.5 × 2.5 × 3.0 cm multinodular mass composed of moderately firm grey-whitish tissues. A nodular mass, with 2.0 cm in diameter, consisting of brown soft tissues (presumably corpus luteum) was also identified (Additional file 1: Figure S1b). No gross abnormality of the right ovary was apparent (Additional file 1: Figure S1a). At cross section, the uterus presented thickened endometrium with multiple and empty cavitary structures of variable dimensions and presence of brown and viscous luminal material.

Representative tissue samples were collected, paraffin-embedded and serial consecutive 2 μm-sections were cut and processed for routine staining (H&E) and immunohistochemistry. The case was examined by two veterinary pathologists (IA and FG) and two human pathologists (JL and CB).

**Fig. 2:**
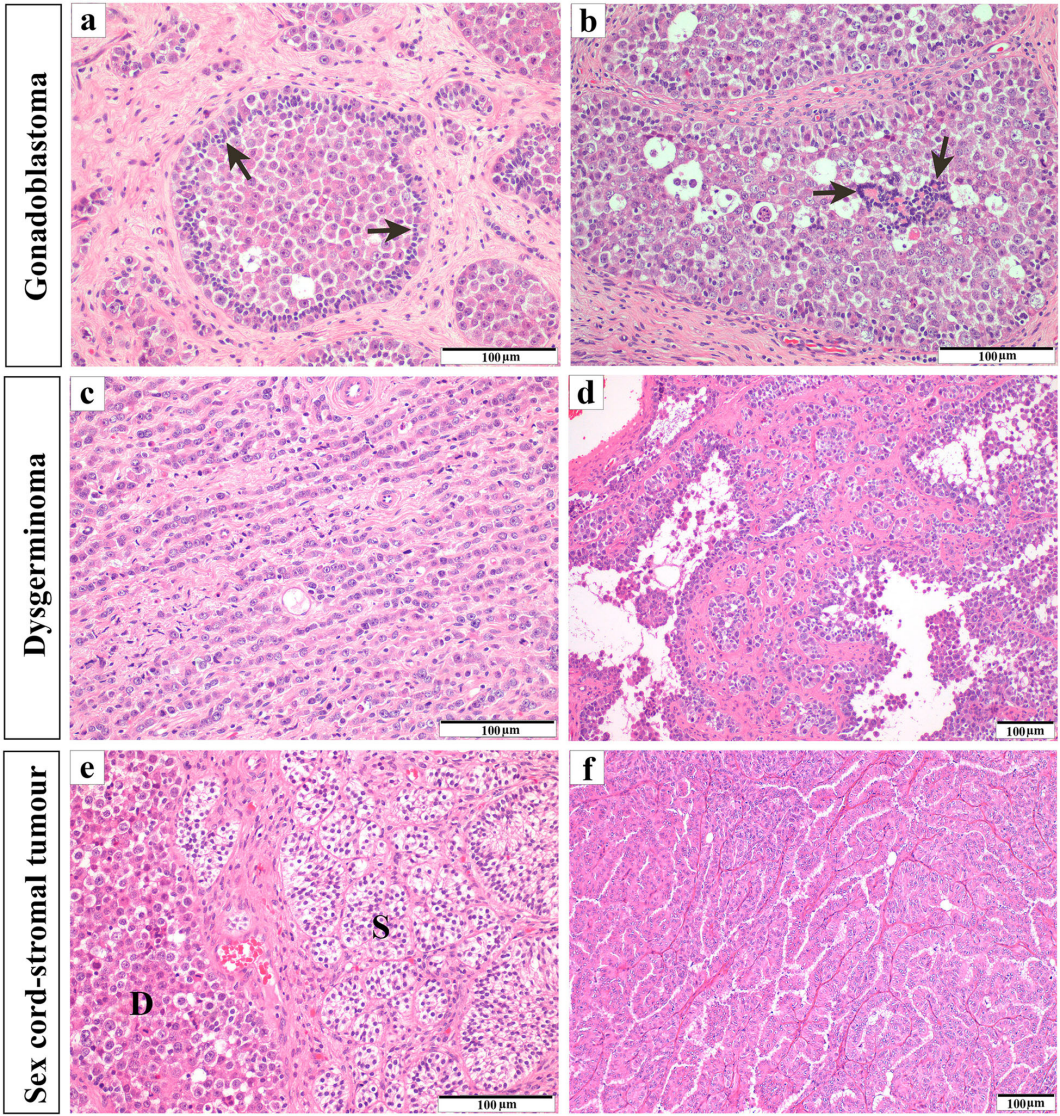
Histological features of the three neoplastic lesion components: Gonadoblastoma, dysgerminoma and sex cord-stromal tumour component (H&E). (**a**) Neoplastic nest composed of germ cells admixed with Sertoli/granulosa-like cells (black arrow), the latter being predominantly in the periphery of the nest (coronal pattern). (**b**) Note Sertoli/granulosa-like cells (black arrow) surrounding small round hyaline bodies within the nest (Call-Exner-like pattern). (**c**) Dysgerminoma cells arranged in cords. (**d**) Note the dysgerminoma pseudo-glandular pattern. (**e**) Proliferation of dysgerminoma (D) and sex cord-stromal neoplastic component resembling a Sertoli tumour (S), the latter being arranged in solid tubules. (**f**) Neoplastic sex cord-stromal cells arranged papillary structures

Microscopically, the normal architecture of the left ovary was diffusely replaced by an irregular, large neoplastic lesion composed of three distinct and intermixed elements: dysgerminoma, mixed germ cell-sex cord-stromal tumour resembling human GB and a proliferative sex cord-stromal component which resembled a Sertoli cell tumour (Fig. 2). The dysgerminoma area consisted of a population of germ cells, arranged in nests, cords (Fig. 2c), microcysts and pseudoglandular spaces (Fig. 2d), surrounded by moderate amounts of fibrous stroma. Some cysts contained eosinophilic proteinaceous material, inflammatory and detached neoplastic cells. The neoplastic cells were large, with round and central nuclei, exhibiting one to three prominent nucleoli and moderate amounts of granular to vacuolated, pale eosinophilic cytoplasm. The GB-like area consisted of neoplastic nests containing germ cells, similar to those described above, intimately admixed with sex cord derivatives resembling Sertoli/granulosa cells (Fig. 2a), arranged in coronal, follicular and Call-Exner-like patterns (Fig. 2b). In the periphery of the neoplasm, minor foci of sex cord-stromal components, arranged in solid tubules (Fig. 2e) and papillary structures (retiform areas), separated by thin connective tissue, were also observed (Fig. 2f). The tubules and papillae were lined by cuboidal to columnar cells, with round to oval nuclei, prominent nucleolus and moderate amount of eosinophilic cytoplasm. Eosinophilic hyaline material within tubular structures was also found. Mitoses (6 per high power field), many of which atypical, were often found in the dysgerminoma component. Multifocal apoptotic bodies were present, as well as macrophages containing cellular debris, giving a “starry sky” appearance. Ovarian stroma adjacent to the neoplasm exhibited several follicles in different stages of development (Additional file 2: Figure S2a) and one corpus luteum (Additional file 2: Figure S2b). No signs of testicular tissue (seminiferous and/or interstitial) were detected in the ovaries. The right ovary was histologically normal.

The uterus showed hyperplastic endometrium with proliferation and cystic dilatation of the endometrial glands, filled with desquamated epithelial cells and scarce inflammatory cells.

### Immunohistochemistry

To support the morphological findings an immunohistochemical study, using a panel of specific antibodies (Table 1), was also made. Briefly, antigen retrieval was performed on dewaxed sections and the Novolink™ Max-Polymer detection system (Novocastra, Newcastle, UK) was used for visualization, according to the manufacturer’s instructions. Slides were then incubated with primary antibodies and appropriate negative and positive controls were included in each slide run (Table 1; Additional file 3: Figure S3).

**Table 1:** Antibodies reagents used in immunohistochemistry

Marker	Type/Clone	Supplier	Dilution	Antigen unmasking	Incubation period	Positive control	Cells of interest
c-KIT (CD117)	Polyclonal	Dako	1:450	RS/WB	ON	Canine mast cell tumour	Germ cells
α-inhibin	Monoclonal/R1	AbDSerotec	1:50	RS/WB	ON	Canine ovarian tissue	Sex cord cells
Pan-cytokeratin	Cocktail AE1/AE3	Menarini	1:300	RS/WB	ON	Canine mammary gland	Epithelial cells
Vimentin	Monoclonal/V9	Dako	1:500	RS/WB	ON	Canine mammary gland	Non-epithelial cells
Oestrogen receptor	Monoclonal/6F11	Novocastra	1:40	RS/WB	ON	Canine endometrial tissue	Epithelial cells
EMA	Monoclonal/E29	Dako	1:20	RS/WB	ON	Canine intestinal tissue	Epithelial cells
Ki-67	Monoclonal/MIB-1	Dako	1:50	RS/WB	ON	Canine lymphoma with high PI	Cells in proliferation

*RS* Retrieval solution, *WB* Water bath, *ON* Overnight, *PI* Proliferative index

**Table 2:** Immunohistochemical staining on the different histological components

Marker	Immunohistochemical reactivity ^b^			
	Gonadoblastoma		Dysgerminoma	Sex cord-stromal tumour
	Germ cells	Sex cord-stromal cells		
c-KIT (CD117)	++	–	++	–
α-inhibin	–	+++	–	−/+++
Pan-cytokeratin	–	–	–	+++
Vimentin	+	++	+	++
Oestrogen receptor	–	–	–	–
EMA	–	–	–	–
Ki-67 PI ^a^	< 1%	–	23%	–

^a^Ki-67 PI (proliferative index): percentage of positive nuclei, determined by counting 1.000 nuclei per 10 high-power fields

^b^Scoring: -, negative; +, weak; ++, moderate; +++, strong

**Fig. 3:**
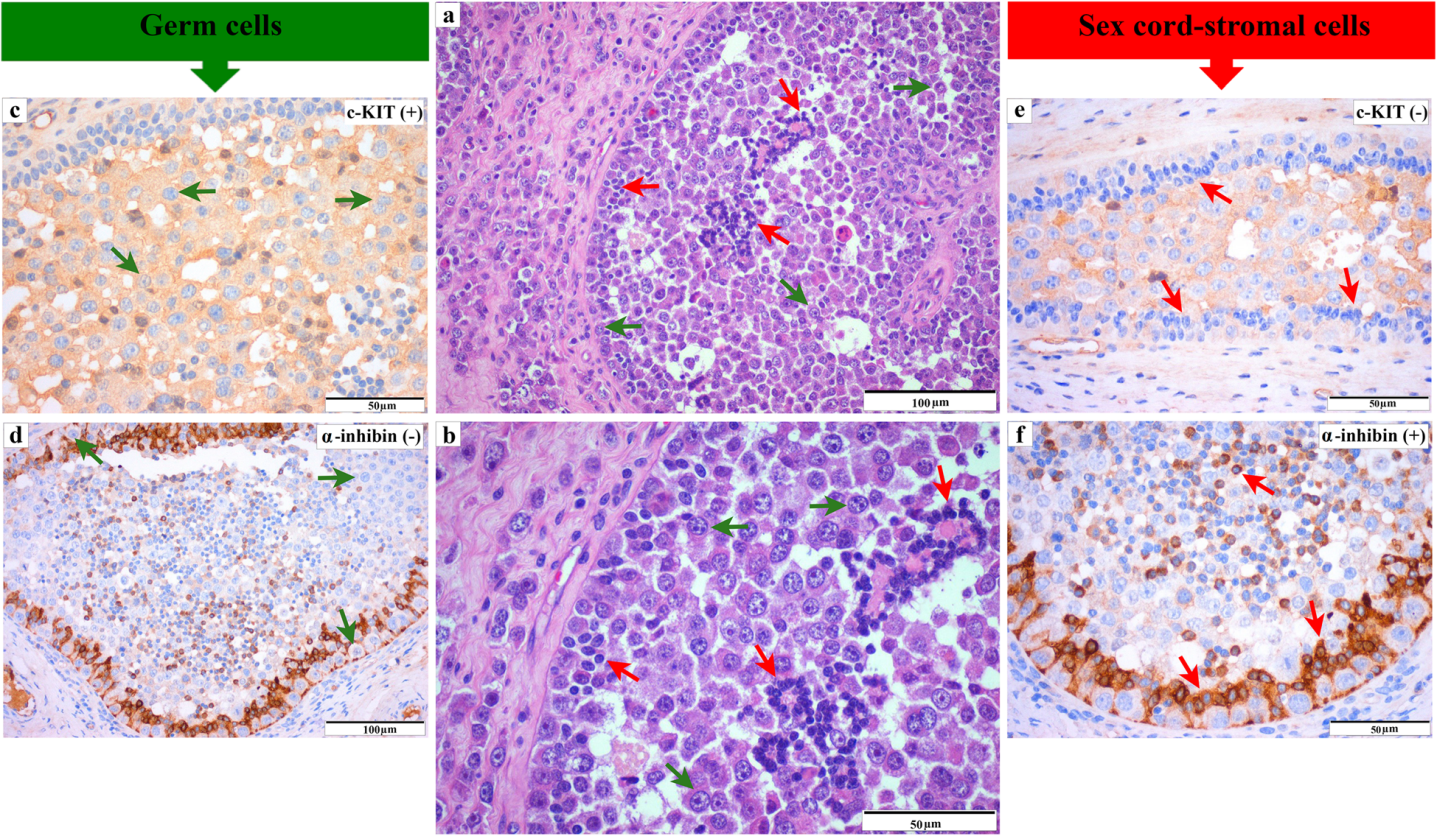
Morphological and immunohistochemical features of the gonadoblastoma. (**a** and **b**) Detail of the neoplastic populations that constitutes the gonadoblastoma. Germ cells (green arrows) and sex cord cells (red arrows) (H&E). Neoplastic germ cells (green arrows) exhibiting moderate immunoreactivity for c-KIT (**c**) and negative for α-inhibin (**d**). (IHQ). Neoplastic sex cord-stromal cells (red arrows) showing strong immunoreactivity for α-inhibin (**f**) and negative for c-KIT (**e**). (IHQ)

**Fig. 4:**
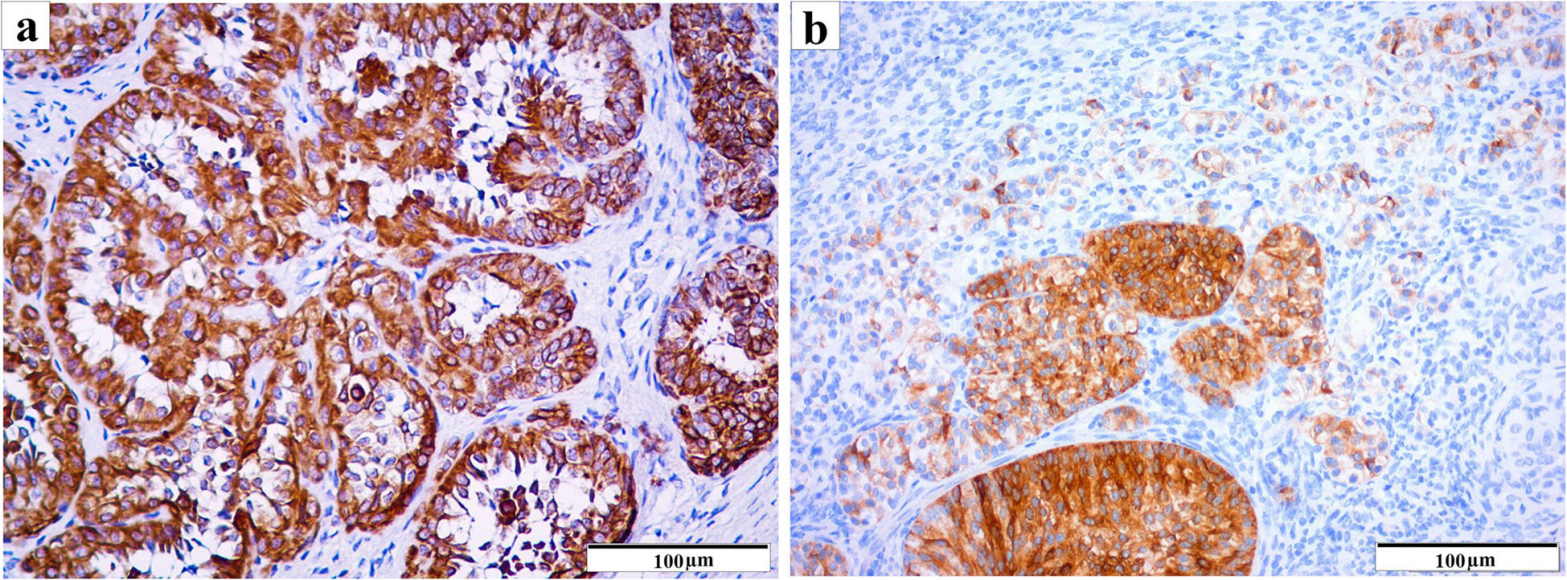
Immunohistochemical features of the sex cord-stromal tumour component. Strong immunoreactivity for AE1/AE3 (**a**) and strong for α-inhibin (**b**). (IHQ)

Immunohistochemical study (Table 2) revealed moderate immunoreactivity for c-KIT (Fig. 3c) in germ cells of GB and dysgerminoma elements, whereas the Sertoli/granulosa-like cells of GB were strongly immunoreactive for α-inhibin (Fig. 3f). The minor foci of sex cord-stromal tumour components were strongly immunoreactive for AE1/AE3 (Fig. 4a), occasionally strong for α-inhibin (Fig. 4b) and negative for oestrogen receptor and EMA. All components were immunoreactive for vimentin, although in the germ cells this immunoreactivity was minimal. Ki-67 immunoreactivity was mainly detected in dysgerminoma cells, with a proliferative index of 23%.

### Chromosome analysis

Conventional cytogenetic and PCR studies were conducted to detect the presence or absence of the Y chromosome. For karyotype analysis, phytohemagglutinin (PHA)-stimulated peripheral whole blood culture was performed for 72 h at 37 °C. The cells were stained with *Giemsa* stain and metaphases analysed under microscope. Additionally, karyotype results were also confirmed via polymerase chain reaction (PCR) based on the differences in the *amelogenin* (*AMEL*) gene, located on both X and Y chromosomes of mammals, as previously described [17]. Briefly, genomic DNA was extracted from the peripheral blood of the dog (Citogene® DNA Blood KIT), according to manufacturer’s instructions. PCR amplification was performed using one pair of primers: DSI-F: 5′-ATAATGACAAAGAAAACATGAC-3’and DSI-R: 5′-CTGCTGAGCTGGCACCAT-3′), designed on the consensus sequences of *AMELX* and *AMELY*, evidencing a single band in the females (215 bp) and two bands (215 and 247 bp) in the males [17].

**Fig. 5:**
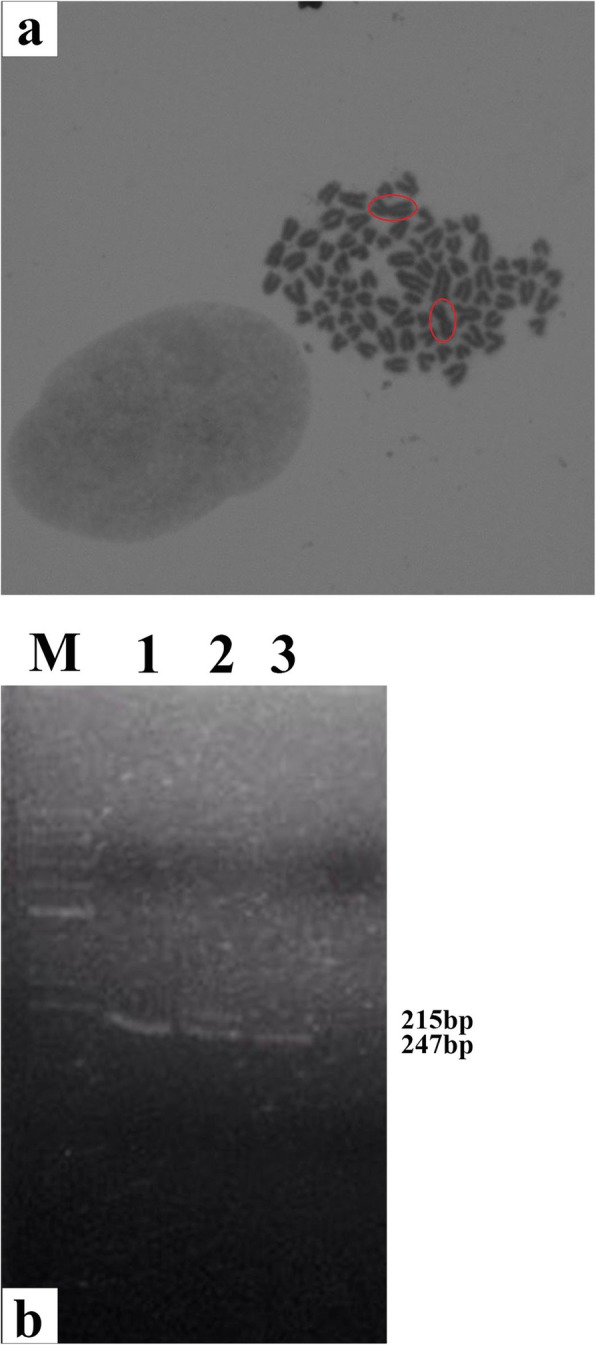
Chromosome analysis of the dog. (**a**) Giemsa-stained metaphase spread: 2n = 78, XX (magnification, 100x). (**b**) Agarose gel electrophoresis of PCR products of canine *AMELX* and *AMELY* gene fragments. M, molecular marker; 1, Dog’s DNA; 2, male DNA; 3, female DNA

The karyotype of the bitch was 78, XX and PCR analysis showed a single band, confirming the absence of the Y chromosome (Fig. 5).

Based on morphological and immunohistochemical findings, a final diagnosis of GB with dysgerminoma and sex cord-stromal tumour proliferation was made. Given the lack of literature regarding this neoplasm at this precise location and affecting this particular animal species, the clinician was alerted to the need to survey the animal and provide careful clinical follow-up. Eleven months after the OVH, the clinical examination revealed no evidence of recurrence nor metastatic disease.

## Discussion

Gonadoblastoma, first described in humans by Scully (1970) [18] is a special type of mixed germ cell-sex cord-stromal tumours, commonly found in children and young adults with abnormal gonadal development. GB is usually a small and solid lesion with bilateral presentation in at least 50% of cases [12]. In dogs, there are only two reported cases of GB, both diagnosed in the testes: one in a differentiated testis of a phenotypic male [14]; and the other consisting of a bilateral GB diagnosed in a male dog with mixed gonadal dysgenesis [15]. In the present case, the ovarian stroma adjacent to the neoplasm had follicles at different stages of development and a corpus luteum, and no evidence of testicular tissue was detected. In addition, the bitch showed no clinical signs suggestive of sex hormone abnormality (such as abnormal genitalia, alopecia, anoestrous or masculinization) and had a history of one successful pregnancy. Taken together, these results indicate that the bitch had apparently normal sexual development and ovarian differentiation.

General morphological and histochemical features of this neoplasm resemble those of human GB (see above). Distinctive trails include the proliferation of the sex cord-stromal elements.

So far, only pure testicular GB were described in dogs [14, 15]. Nevertheless, a testicular GB with concomitant seminoma was previously described in a pet domestic rabbit [16]. GB has been considered an in situ germ cell tumour that commonly progresses to invasive germinoma or another type of malignant germ cell tumour [19]. Proliferation of sex cord-stromal components associated with GB has been rarely reported in humans [19–21] and has never been recorded in animals. In this case, the GB was associated with dysgerminoma and an area of proliferated sex cord-stromal elements resembling Sertoli cell tumour, suggesting that the sex cord elements of GB may also progress to an independent sex cord-stromal tumour.

The histological subtyping of ovarian neoplasms represents a challenge for pathologists and usually needs additional studies, such as immunohistochemistry.

c-KIT protein (CD117) is the receptor for stem cell factor, being responsible for proper migration of primordial germ cells during development. It is present only in immature germ cells and is highly expressed in early stages of germ cell development [22]. c-KIT is the most useful diagnostic marker for human dysgerminoma [23]. Previous studies have demonstrated c-KIT expression in germ cells of human and canine GB, but not in the sex cord-stromal elements. The latter are frequently immunoreactive for α-inhibin, whereas germ cells are negative [15, 19, 24]. Thus, α-inhibin, a gonadal glycopeptide that suppresses follicle-stimulating hormone, is a useful marker of canine [25] and human sex cord-stromal tumours [26].

Vimentin and cytokeratin are expressed in many ovarian epithelial cell tumours [3, 27], germ cell tumours [3] and sex cord-stromal tumours [3], although with variable degrees of immunoreactivity. In contrast, EMA is consistently negative in ovarian sex cord-stromal tumours [28], but positive in most epithelial cell tumours [29, 30]. The triad of EMA negative, α-inhibin positive and calretinin positive is useful for the distinction between human sex cord-stromal tumours and epithelial tumours, such as endometrioid carcinomas with sex cord-like patterns [26]. Also in dogs, positive α-inhibin and negative EMA are useful tools for differential diagnosis of ovarian sex cord-stromal tumours [31].

In the current case, the immunohistochemical expression of c-KIT in germ cells and α-inhibin in sex cord-stromal cells supports the histological diagnoses of GB and dysgerminoma. The positive expression for α-inhibin and the negative result for EMA in the area with sex cord-stromal components also emphasizes the histological diagnosis of sex cord-stromal tumour.

GB is mostly found in patients with gonadal dysgenesis containing Y chromosome. In our case, the bitch presented normal 78, XX karyotype and no evidence of Y chromosome in PCR study, suggesting this GB might be unrelated to Y chromosome. To the best of our knowledge, this is the first report of cytogenetic studies in a veterinary species with GB. Thus, we performed a review of the human literature for relevant articles, regarding GB with normal 46, XX karyotype, published between 1990 and 2018. There were 16 cases reported, all occurring in normal phenotypic females, most of them fertile and with history of successful pregnancies. Most were unilateral and were associated with a germ cell tumour, commonly dysgerminoma (Table 3).

**Table 3:** Gonadoblastoma with normal 46, XX karyotype: data from the human literature

Author (Reference)	Age (years)	Coexistence	Bilaterality	Karyotype	Clinical presentation	Treatment
Elemenoglou et al., 1990 [32]	NA	NA	NA	46, XX	Pregnant	NA
Erhan et al., 1992 [33]	26	DYG	No	46, XX	Pregnancy+ adnexal mass	H + BO+ CHT
Obata et al., 1995 [34]	10	DYG and MGCT^a^ (DYG+ YSC)	Yes	46, XX	Abdominal pain	BO+ CHT
Zhao et al., 2000 [35]	27	MGCT (COC+ EC+ YST+ IT)	No	46, XX	Abdominal mass	UO+ CHT+ H+ UO+ L+ O+ HRT
Kim et al., 2003 [19]	33	DYG and SCST	No	46, XX	Abdominal mass	UO+ CHT
Kim et al., 2003 [19]	30	DYG	No	46, XX	Abdominal Pain	UO
Erdemoglu and Ozen, 2007 [36]	19	EST	No	46, XX	Abdominal mass + pain	UO
Gorosito et al., 2010 [37]	17	DYG	No	46, XX	Pregnancy+ ovarian mass	CHT+ H+ UO
Yilmaz et al., 2010 [38]	20	DYG	Yes	46, XX	Increased abdominal girth	BO+ CHT+ RT
Koo et al., 2011 [39]	34	DYG	No	46, XX	Vaginal bleeding	UO+ L+ CHT
Young et al., 2012 [40]	28	DYG + SCTAT and DYG^a^	No	46, XX	Abdominal mass	BO+ O+ CHT+ H
Esin et al., 2012 [41]	15	DYG	No	46, XX	Irregular vaginal bleeding+ pelvic pain	UO
Kanagal et al., 2013 [42]	14	DYG	No	46, XX	Abdominal mass	UO+ CHT
Kulkami et al., 2016 [43]	20	DYG	No	Absence of Y in FISH	Abdominal pain	UO
Gru et al., 2017 [44]	24	MGCT (DYG + ST)	No	46, XX	Incidental finding	UO
Arafah et al., 2018 [45]	19	MGCT (YSC+ COC)	No	46, XX	Abdominal heaviness+ increased abdominal pain	UO+ O+ CHT

*NA* Not available, *DYG* Dysgerminoma, *YSC* Yolk sac tumour, *MGCT* Mixed germ cell tumour, *COC* Choriocarcinoma, *EC* Embryonal carcinoma, *IT* Immature teratoma, *SCST* Sex cord-stromal tumour, *EST* Endodermal sinus tumour, *SCTAT* Sex cord-stromal tumour with annular tubules, *ST* Spermatocytic tumour-like, *FISH* Fluorescence in situ hybridization, *UO* Unilateral oophorectomy, *H* Hysterectomy, *BO* Bilateral oophorectomy, *CHT* Chemotherapy, *L* Lymphadenectomy, *O* Omentectomy, *HRT* Hormonal replacement therapy, *RT* Radiotherapy

^a^contralateral ovary

In humans the prognosis of either pure GB or GB associated with dysgerminoma, when treated, is excellent; however, the presence of other types of germ cell tumours makes the prognosis usually less favourable [12]. The present GB was overgrown by dysgerminoma and a small area of sex cord-stromal tumour components. Given the rarity of similar findings in both human and animals, the biological behaviour of this entity is not fully understood. Nevertheless, considering the absence of vascular or lymphatic invasion, ovarian surface involvement and the low proliferative index, a good prognosis was suspected. Additionally, no evidence of invasive or metastatic disease was found during 11 months of follow-up which suggests a benign clinical course for this neoplasm.

## Conclusions

This is the first report of an ovarian GB in a bitch. Despite being a phenotypically and cytogenetically normal bitch, the neoplasm exhibited morphological and immunohistochemical features resembling human GB. Accordingly, GB should be considered as a differential diagnosis for ovarian tumours composed of germ cells and sex cord-stromal cells in domestic animals and immunohistochemistry study may be performed to support this diagnosis.

## Additional files

Additional file 1: **Figure S1.** Macroscopic findings. Gross aspect of the normal (a) and affected ovary (b). Note the multinodular appearance and the heterogenous colour of the affected ovary. (JPG 227 kb)

Additional file 2: **Figure S2.** Histological sections from the adjacent normal ovarian tissue**.** (a) In the ovarian stroma are visible follicles at different stages of development. PF, primary follicle; PoF, primordial follicle. (b) Portion of adjacent corpus luteum (CL). (JPG 1086 kb)

Additional file 3: **Figure S3.** Validation of immunohistochemical expression. (a, c, e, g, i, k, m) Immunohistochemical expression in the different components of the adjacent normal ovarian tissue. (b, d, f, h, j, l, n) Positive control tissues used. (b) Canine mast cell tumour. (d) Canine ovarian tissue. (f and h) Canine mammary gland. (j) Canine endometrial tissue. (l) Canine intestinal tissue. (n) Canine lymphoma with high PI. (JPG 2451 kb)

## Data Availability

The authors declare that the data supporting the findings of this study are available within the article.

## Abbreviations

AMELX-Y: *amelogenin* X-Y
CRT: Capillary repletion time
DNA: Deoxyribonucleic acid
EMA: Epithelial membrane antigen
GB: Gonadoblastoma
H&E: Haematoxylin and Eosin
MGCSCST: Mixed germ cell-sex cord-stromal tumour
OVH: Ovariohysterectomy
PCR: Polymerase chain reaction
PHA: Phytohemagglutinin
WHO: World Health Organization
α-inhibin: Alpha-inhibin
