# Supplementary figures and images for: Canine ovarian gonadoblastoma with dysgerminoma overgrowth: a case study and literature review
Source: J Ovarian Res. 2019 Sep 23;12:89. doi: 10.1186/s13048-019-0561-x (PMC6757443; doi:10.1186/s13048-019-0561-x)

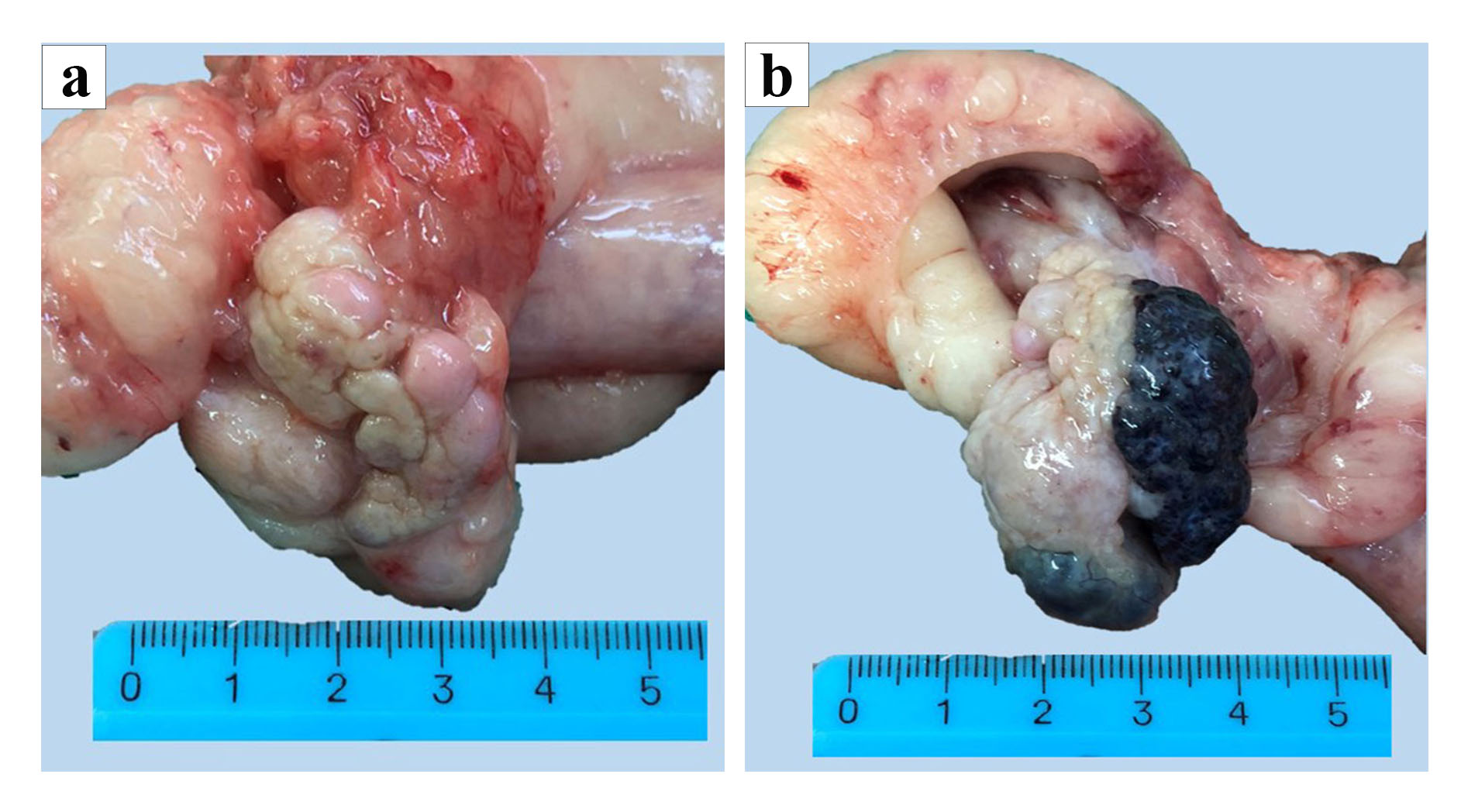

Supplement: Supplementary file 1 — Figure S1. Macroscopic findings. Gross aspect of the normal (a) and affected ovary (b). Note the multinodular appearance and the heterogenous colour of the affected ovary. (JPG 227 kb) [file 13048_2019_561_MOESM1_ESM.jpg]

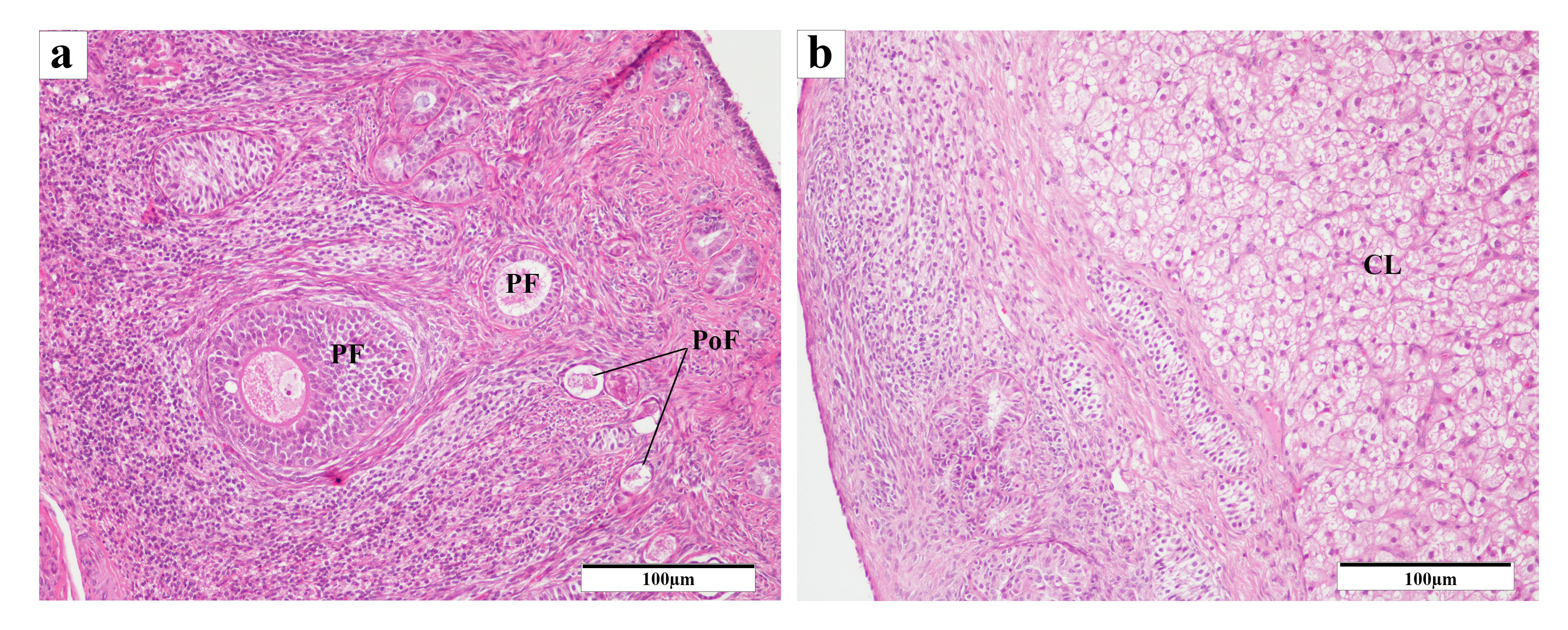

Supplement: Supplementary file 2 — Figure S2. Histological sections from the adjacent normal ovarian tissue. (a) In the ovarian stroma are visible follicles at different stages of development. PF, primary follicle; PoF, primordial follicle. (b) Portion of adjacent corpus luteum (CL). (JPG 1086 kb) [file 13048_2019_561_MOESM2_ESM.jpg]

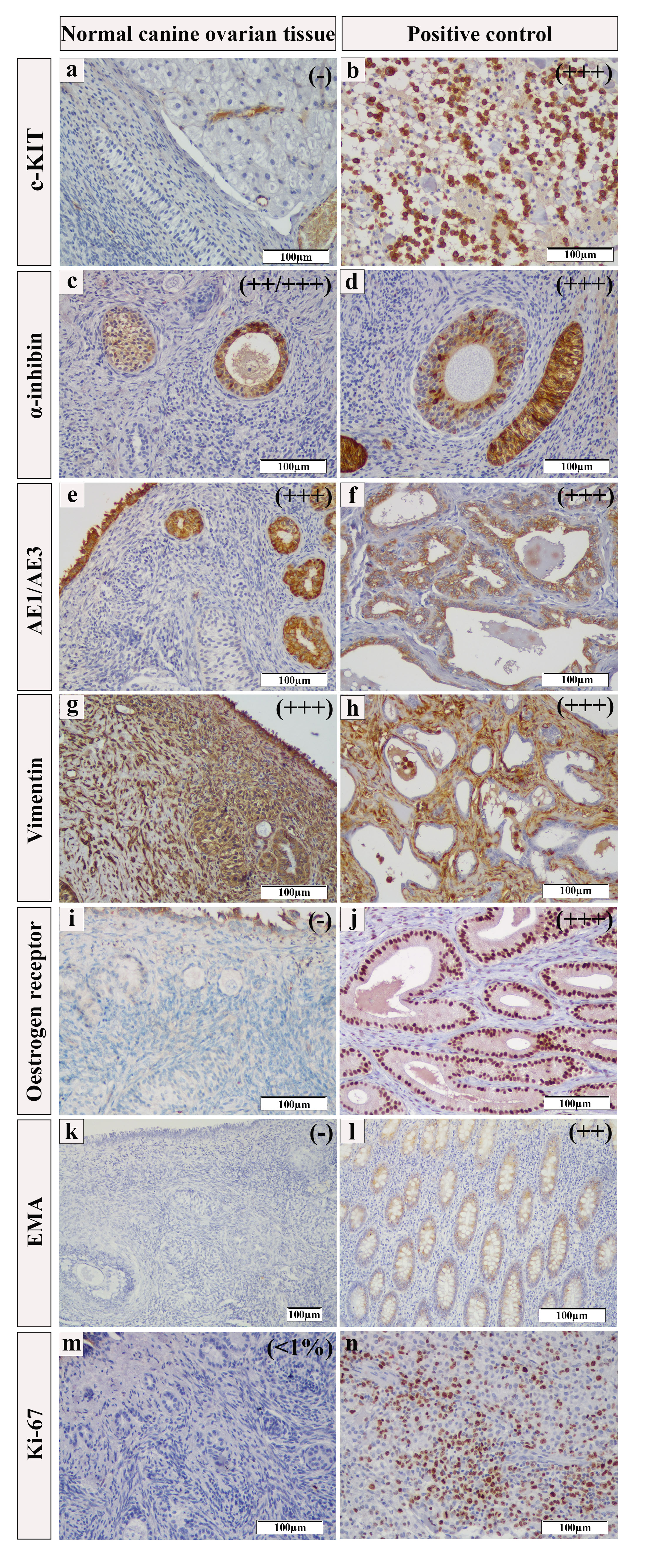

Supplement: Supplementary file 3 — Figure S3. Validation of immunohistochemical expression. (a, c, e, g, i, k, m) Immunohistochemical expression in the different components of the adjacent normal ovarian tissue. (b, d, f, h, j, l, n) Positive control tissues used. (b) Canine mast cell tumour. (d) Canine ovarian tissue. (f and h) Canine mammary gland. (j) Canine endometrial tissue. (l) Canine intestinal tissue. (n) Canine lymphoma with high PI. (JPG 2451 kb) [file 13048_2019_561_MOESM3_ESM.jpg]
